# Towards the Definition of the Molecular Hallmarks of Idiopathic Membranous Nephropathy in Serum Proteome: A DIA-PASEF Approach

**DOI:** 10.3390/ijms241411756

**Published:** 2023-07-21

**Authors:** Paolo Previtali, Lisa Pagani, Giulia Risca, Giulia Capitoli, Eleonora Bossi, Glenda Oliveira, Isabella Piga, Antonella Radice, Barbara Trezzi, Renato Alberto Sinico, Fulvio Magni, Clizia Chinello

**Affiliations:** 1 Proteomics and Metabolomics Unit, School of Medicine and Surgery, University of Milano-Bicocca, 20854 Vedano al Lambro, Italy; p.previtali1@campus.unimib.it (P.P.); lisa.pagani@unimib.it (L.P.); eleonora.bossi@unimib.it (E.B.); g.oliveira1@campus.unimib.it (G.O.); isabella.piga@unimib.it (I.P.); fulvio.magni@unimib.it (F.M.); 2 Bicocca Bioinformatics Biostatistics and Bioimaging Centre—B4, School of Medicine and Surgery, University of Milano-Bicocca, 20854 Vedano al Lambro, Italy; g.risca@campus.unimib.it (G.R.); giulia.capitoli@unimib.it (G.C.); 3 Microbiology Institute, ASST (Azienda Socio Sanitaria Territoriale) Santi Paolo e Carlo, 20142 Milan, Italy; antonella.radice@asst-santipaolocarlo.it; 4 Department of Medicine and Surgery, University of Milano Bicocca and Nephrology, 20900 Monza, Italy; barbara.trezzi@unimib.it (B.T.); renato.sinico@unimib.it (R.A.S.); 5 Dialysis Unit, ASST-Monza, Ospedale San Gerardo, 20900 Monza, Italy

**Keywords:** membranous nephropathies, proteomics, mass spectrometry, DIA-PASEF, serum

## Abstract

Idiopathic membranous nephropathy (IMN) is a pathologically defined disorder of the glomerulus, primarily responsible for nephrotic syndromes (NS) in nondiabetic adults. The underlying molecular mechanisms are still not completely clarified. To explore possible molecular and functional signatures, an optimised mass spectrometry (MS) method based on next-generation data-independent acquisition combined with ion-mobility was applied to serum of patients affected by IMN (n = 15) or by other glomerulopathies (PN) (n = 15). The statistical comparison highlighted a panel of 57 de-regulated proteins with a significant increase in lipoprotein-related proteins (APOC1, APOB, APOA1, APOL1 and LCAT) and a substantial quantitative alteration of key serpins (including A4, D1, A7, A6, F2, F1 and 1) possibly associated with IMN or NS and podocyte stress. A critical dysregulation in metabolisms of lipids (e.g., VLDL assembly and clearance) likely to be related to known hyperlipidemia in IMN, along with involvement of non-classical complement pathways and a putative enrolment of ficolin-2 in sustaining the activation of the lectin-mediated complement system have been pinpointed. Moreover, mannose receptor CD206 (MRC1-down in IMN) and biotinidase (BTD-up in IMN) are able alone to accurately distinguish IMN vs. PN. To conclude, our work provides key proteomic insights into the IMN complexity, opening the way to an efficient stratification of MN patients.

## 1. Introduction

Membranous nephropathy (MN) is a kidney disease characterised by the thickening of the glomerular basement membrane (GBM) together with the antigen–antibody immune complex formation in the subepithelial area of GBM [1]. MN is the cause of about 20% of adult nephrotic syndromes (NS) and it is the second leading cause of primary glomerulonephritis and end-stage renal disease (ESRD). Symptoms include severe proteinuria, oedema, hypoalbuminemia and hyperlipidemia [2].

In developed countries, about 75% of MN cases have no clear secondary causes and are referred to as idiopathic membranous nephropathy (IMN) or primary membranous nephropathy (PMN), while 20–25% are secondary membranous nephropathy (SMN) and are due to different conditions, such as infections, malignancies, systemic autoimmune disease and drug toxicity [3]. The gold standard for IMN and SMN diagnosis is the renal biopsy: the pathologic hallmark of MN is the presence of subepithelial immune complex deposits which can be detected by immunofluorescence and electron microscopy [4,5]. Owing to its invasive nature, the use of kidney biopsies is limited for repeated applications in clinical diagnoses. There is a great significance to exploring sensitive, specific and non-invasive biomarkers for the diagnosis and treatment of MN. Glomerulonephritis has different pathogenic mechanisms and requires distinct therapies; thus, non-invasive molecular diagnosis should be very effective in refining medical decision-making and patient care. For instance, blood biomarkers that improve risk stratification of IMN at the time of diagnosis would allow earlier and tailored treatment with immunosuppressants and avoid inappropriate or harmful treatment in patients who may experience spontaneous remission, or in patients with MN secondary to hepatitis B or hepatitis C infections that have to be treated by antiviral drugs [6].

The search for antigens in IMN was unsuccessful for many years but, in 2009 and 2014, PLA2R and THSD7A autoantigens were, respectively, identified by using an approach based on microdissection of human glomeruli, proteomic technology and mass spectrometry. The first major autoantigen consists of the muscle-type PLA2R [7]. In particular, circulating autoantibodies to PLA2R (mainly belonging to the IgG4 subclass) were detected in about 70% of patients with IMN. The second autoantigen is THSD7A and the circulating autoantibodies against this protein were detected in 5–10% of patients negative for anti-PLA2R [8]. Recently, a great number of new target antigens have been identified in MN using the MS technique on both affected tissue and urine samples, such as exostatin 1/2 (EXT1/EXT2), NELL-1, semaphorine-3B (Sema-3B), neural cell adhesion molecule 1 (NCAM-1), high-temperature recombinant protein A1 (HTRA1) and protocadherin 7 (PCDH7). IMN-related target antigens can be detected in 80% of cases, of which 70–80% are PLA2R positive, approximately 1% are PLA2R and THSD7A double positive, and 20–30% are negative for PLA2R, including THSD7A, NELL-1, Sema-3B and THRA-related IMN (approximately 13%). However, 10–20% of IMN-related autoantigens remain to be discovered [1].

For these reasons, several MS approaches have been developed to further characterise the proteomic hallmarks of IMN, with the aim of shedding light on the molecular mechanisms underlying the pathology. The application of proteomics to MN has been primarily performed in urine and kidney biopsy samples. Choi et al. used an LC-MS/MS approach to study the urine proteome of patients affected with minimal change disease (MCD), focal segmental glomerulosclerosis (FSGS) and membranous nephropathy (MN), leading to the identification of 228 urine proteins, of which 22 were differentially expressed between the groups, thus representing putative novel biomarkers [9]. Chinello et al. have explored the role of aberrant Fc glycosylation of IgG in IMN as the trigger of the autoimmune response at the base of its etiopathogenesis by conducting an MS-based N-glycan profiling on serum samples [10]. Newer techniques, such as matrix-assisted laser desorption/ionisation mass spectrometry imaging, are also being applied for biomarker discovery with promising results in kidney biopsies of patients with MN [11]. Daniel A. Muruve et al. also tried to identify serum protein biomarker signatures associated with MCD and MN pathogenesis by using a targeted approach consisting of the application of the SOMAscan^®^ aptamer-based proteomic technique [6].

Despite these advancements, no significant high-throughput untargeted proteomics studies have been conducted on IMN, especially in blood samples. Discovery proteomics on blood and its derivatives, such as serum and plasma, may play a critical role in providing deeper insights to better understand the molecular and functional signature of the pathology and, consequently, to develop novel diagnosis and therapy protocols that could enhance patient outcomes. Human biofluids are indeed widely employed in many clinical tests and analyses, due to the low cost and minimally invasive sample collection and processing; however, many challenges remain as a consequence of their unique characteristics and complex composition [12]. Its application may indeed be hampered by several factors including the interference ascribed to abundant proteins (albumin and immunoglobulins) profusely present in blood which prevent the identification of low-abundant proteins that may be highly clinically and biologically relevant [13,14].

Emerging MS technologies may have the potential to overcome these challenging aspects and limitations in blood protein identification and quantification, enabling a direct analysis of undepleted samples, allowing for the exploration of whole proteomes and their variations [15,16]. Recent improvements in MS instrumentation and software have enabled alternative workflows, known as data-independent acquisition (DIA) methods, able to enhance robustness, data consistency and quantitative accuracy. In particular, a recent DIA acquisition method, called DIA-PASEF (parallel accumulation serial fragmentation), based on a trapped ion mobility separation (TIMS) device, permits integration of an ion mobility separation of proteomic samples within the mass spectrometer, allowing a significant gain in sensitivity, increasing the quality of spectra and clearing the way for a deeper characterization of the proteomes [17].

Based on this context, the focus of this pilot study is aimed to investigate the blood proteome of patients affected by IMN and patients affected by other types of nephropathies (PN) in order to identify possible molecular and functional signatures specific to IMN. To this end, a tailor-made data-independent acquisition-parallel accumulation serial fragmentation (DIA-PASEF) method was optimised and applied to undepleted serum samples of PLA2R antibody-positive patients with IMN and of individuals with nephrotic syndromes not ascribable to IMN. They were selected from the cohort with the goal of gaining a step towards the unravelling of the molecular complexity defining this disease.

## 2. Results

The study of the proteome involves large-scale detection, identification and characterisation of proteins that together form the complex signalling networks constituting the active cellular proteome, critical for the development of effective diagnostic and prognostic tools across many diseases [18]. The DIA-PASEF acquisition method was selected as the method of choice, as it combines the PASEF principle for the MS/MS analysis with the advantages of DIA-MS to enhance sensitivity and reduce spectral complexity, ensuring a high-throughput characterisation of the samples [19]. Herein, a tailor-made DIA-PASEF method was applied to quali-quantitatively portray undepleted serum proteome collected from 15 IMN patients and 15 gender- and age-matched nephropathic subjects having a similar presentation but a different aetiology (PN) (Table 1).

### 2.1. Proteome Variations Associated with IMN

The results of the DIA-PASEF library-free data analysis on the entire cohort in terms of identifications per run and sample correlation are illustrated in Figure S1. An average CV of 3% in terms of the number of protein groups identified between technical duplicates was calculated and a strong correlation between technical replicates was shown (Figure S1b). The MS/MS analysis identified a total of 4477 distinct peptides, resulting in the identification and quantification of 588 protein groups (652 nonredundant protein isoforms). Among them, 467 protein groups (510 protein isoforms) were identified and quantified with at least two significant peptides and considered for the label-free relative quantification (Tables S1 and S2).

**Figure 1:**
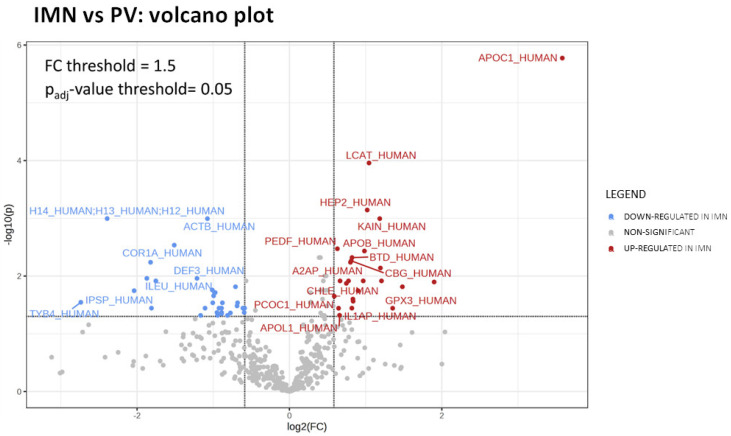
Volcano plot. Volcano plot showing the differentially expressed protein groups between IMN and PN groups. FC is set to 1.5 and p_adj_-value at least < 0.05. Blue = downregulated proteins; red = upregulated proteins; grey = nonsignificant and/or unvaried proteins.

To evaluate the presence of a molecular and functional signature specific to IMN serum samples, a comparison between the two studied groups of patients was performed. A total of 57 serum protein groups were found to be significantly different in abundance in IMN patients versus PN controls. Among these differentially regulated proteins (DEPs), 25 were upregulated in IMN, whereas the remaining 32 showed a significant reduction in their serum concentration in IMN patients (Table 2 and Table S2). As depicted in the volcano plot (Figure 1), apolipoprotein C1 (APOC1) was the most significantly upregulated in IMN, together with phosphatidylcholine-sterol acyltransferase (LCAT), heparin cofactor-2 (HEP2), kallistatin (KAIN) and apolipoprotein B-100 (APOB) (FC ≥ 1.97; p_adj_-value < 0.003).

The dataset was further explored by performing a hierarchical clustered analysis (HCA) using heatmap data visualisation. Data autoscaling was applied, and data were clustered in the two groups (IMN and PN) by using the top 76 most statistically significant features (p_adj_-value < 0.05 using a *t*-test). Interestingly, the HCA results showed that the two sets of proteins (upregulated and downregulated) separated IMN and PN groups into two distinct clusters, with the exception of only a few samples (IMN v30, IMN v9, PN2 and PN17) that were clustered as outliers under the tree of the opposite group. Despite this slight discrepancy, also explained by the possible biological variability between samples, this outcome further confirmed the presence of a serum molecular signature specific to IMN patients and different for patients with other nephrotic syndromes (Figure S2).

### 2.2. Dysregulation of Lipid Metabolism and Immune System Modulation in IMN: In Silico Functional Signatures

To gain a more complete overview of the alterations and the related molecular impacts occurring in the idiopathic form of membranous nephropathy and provide insights into key pathways elicited in the studied population, an in silico functional enrichment analysis was performed based on the 57 DEPs. Details about this annotation pipeline were reported in Figure S3.

Considering only the pathways and the biological processes involving proteins upregulated in IMN, the critical role of lipoproteins and their metabolism (both in terms of assembly and clearance), with the significant involvement of *APOC4*, *APOC1*, *LCAT* and *APOB* genes has been highlighted by the enrichment analysis (Figure S3a,c).

Similarly, the panel of the 32 downregulated proteins in IMN (and thus upregulated in PN) was used to perform the functional enrichment analysis, in order to explore possible depleted and/or dampened pathways. In general, the analysis showed significant enrichment in terms mainly belonging to the “immune system” and “disease” nodes (p_adj_-value < 0.05), as summarised in Figure S3b,c. In particular, PN serum samples displayed a strong activation of the innate immune system, including the activation and regulation of the complement system and other humoral responses (such as FCGR activation and Fgamma receptor (FCGR) dependent phagocytosis).

Idiopathic MN is an organ-specific autoimmune disease involving the kidney; thus, the immune system plays a central role in the pathogenesis and progression of the pathology [2]. To better define the possible involvement of the immune response mechanisms in IMN and gain a more complete landscape of the possible functional alterations connected to the disease, a robust but less stringent statistical sieve was adopted. A total of 100 serum proteins were found to be significantly different in their abundances in the IMN/PN comparison (Figure S4). Consistent with the previous outcome, other serpins (serpin A11 (SPA11, coded by *SERPINA11* gene), alpha-1-antitrypsin (A1AT, coded by *SERPINA1* gene) and plasma protease C1 inhibitor (IC1, coded by *SERPING1* gene) were found to be significantly varied in their concentration. In particular, SPA11 and A1AT were overexpressed in IMN sera, contrariwise to IC1 that resulted downregulated.

**Figure 2:**
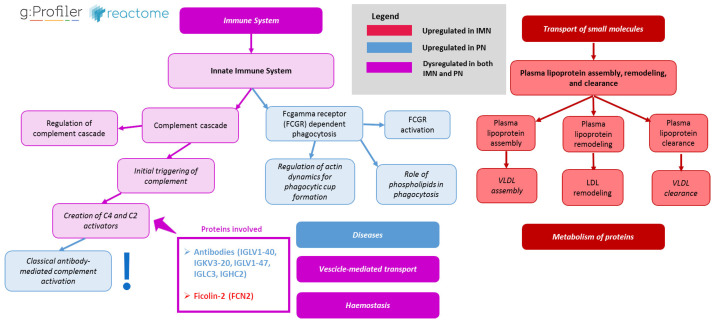
Summary of the most relevant deregulated pathways. This graph shows the most relevant processes which are upregulated in IMN (in red), in PN (in blue) and in both groups (in purple), with a particular focus on complement cascade involvement (g:Profiler [20]). The graph is illustrated following the hierarchical clusterisation reported in Reactome database.

**Figure 3:**
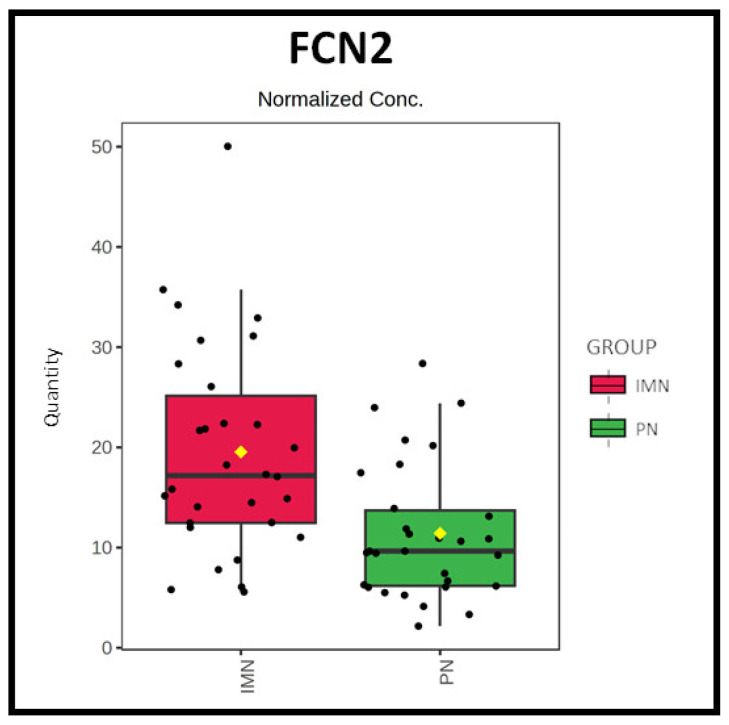
Boxplot of FCN2. Boxplot of FCN2, which was found to be significantly upregulated in IMN (p_adj-_value < 0.05).

This panel of 100 differentially expressed proteins was subsequently employed for the functional enrichment analysis in the g:Profiler platform (Figure S5) [20]. The outcome of the analysis is illustrated in Figure S5. As expected, the use of a larger dataset enabled a broader perspective on the functional modulation in disease, including immune system-related pathways. Consistent with previous results, the analysis confirmed an IMN-specific enrichment in lipoproteins-related pathways for the upregulated panel of proteins and an enrichment in immune- and disease-related pathways for the PN-upregulated panel of proteins, including the strong involvement of the innate immune system and the pathways related to complement cascade and FCGR-related responses. However, interestingly, it was also possible to observe a specific enrichment for the classical antibody-mediated complement activation only in the PN group and not in the IMN group. This result was partially expected since the classical pathway is not usually activated in most cases of primary MN [10]. Moreover, both groups showed enrichment in the “creation of C4 and C2 activators’’ pathway, fundamental for the initial triggering of the complement cascade (Figure 2). Nevertheless, the genes involved in this biological function differed between the two groups. In particular, in PN cases, the activation of C4 and C2 seems to be mainly associated with the over-expression of the immunoglobulins (*IGLV1-40, IGKV3-20, IGLV1-47, IGLC3* and *JCHAIN* genes), whereas in IMN, the activation of these factors appeared related to the up-regulation of one key gene, that is *FCN2*, whose abundance was also seen as significantly increased in the first more restrictive statistical analysis (FC = 1.7; p_adj_-value  = 0.007) (Figure 3).

### 2.3. Proteomic Tree Clustering: MRC1 and BTD

**Figure 4:**
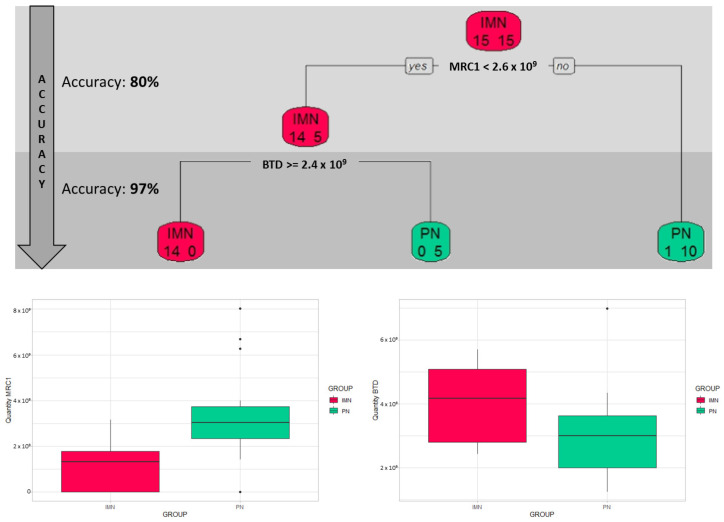
Decision tree. The decision tree shows the proteins which are able to discriminate IMN patients from PN ones and their cut-off. In each box are specified the following values: the name of the more frequent class (IMN or PN) and the number of cases belonging to the two classes, on the left are IMN cases and on the right are the PN cases. Below, the boxplots of MRC1 and BTD for each group are shown. The 5 PN clusterised with IMN in the first step include PN10 (IgAN), PN14 (IgAN), PN2 (FGS), PN3 (MCD/FSGS) and PN7 (MPGN).

The specificity and the impact of the molecular signatures associated with IMN disease were more deeply explored, applying an additional supervised analysis based on a classification tree (Figure 4).

The first branch key factor, able to separate IMN patients from PN ones, emerged as MRC1 protein (*MRC1*—macrophage mannose receptor 1), with a cut-off of 2.6 × 10^9^ and an accuracy of 80%. In the first step, about 93% of IMN and 67% of PN patients are classified in the right group, considering the MRC1 cut-off.

As a second step, BTD protein (*BTD*—biotinidase) can separate the remaining PN patients from IMN ones with a cut-off of 2.4 × 10^9^ reaching a final accuracy of 97% (confidence interval: 83–99%).

Considering the IMN group as the positive condition, the decision tree reaches a sensitivity of 93% (confidence interval: 79–98%) and a very high value of specificity equal to 100% (confidence interval: 89–100%).

The decision tree highlights two proteins (MRC1 and BTD) which, among all the proteins which resulted deregulated in the two groups, could be possible candidates in helping to discriminate IMN disease from other types of nephropathies.

## 3. Discussion

The present work aimed at investigating the molecular and functional hallmarks for idiopathic membranous nephropathy (IMN) by using a novel DIA-PASEF acquisition method for the nLC-MS/MS (nano liquid chromatography-tandem mass spectrometry)-analysis of undepleted serum samples collected from patients.

A significant dysregulation of lipoprotein-related proteins in IMN sera compared to PN controls was enlightened. Lipoproteins are lipidated protein particles that are needed to transport to various tissues hydrophobic substances (such as lipids and vitamins), through the hydrophilic environment of plasma. They can be classified on the basis of their hydrated density—in ascending order—into chylomicrons, very-low-density lipoproteins (VLDL), intermediate-density lipoproteins (IDL), low-density lipoproteins (LDL) and high-density lipoproteins (HDL). Their core typically contains hydrophobic molecules, such as triglycerides (TG) and cholesteryl esters, whereas their surface consists of either hydrophilic or amphipathic substances, such as phospholipids and cholesterol, as well as apolipoproteins. Apolipoproteins are fundamental since they serve as templates for the assembly of lipoproteins, maintain their structure and direct their metabolism by binding membrane receptors and regulating several enzyme activities (such as lipoprotein lipase (LPL), hepatic triglyceride lipase (HTGL), lecithin-cholesterol acyltransferase (LCAT) and cholesteryl ester transfer protein (CETP)) [21].

**Figure 5:**
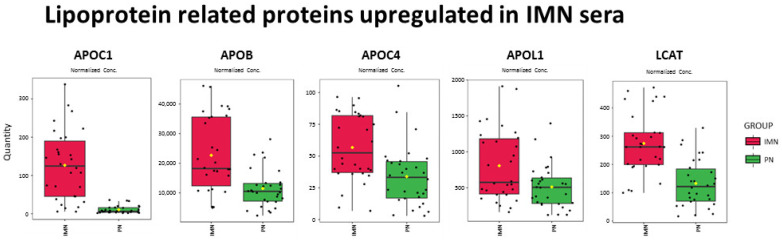
Lipoprotein-related proteins upregulated in IMN. Boxplots of the lipoprotein-related proteins (APOC1, APOB, APOC4, APOL1 and LCAT) found to be significantly upregulated (p_adj_-value < 0.05) in IMN serum samples. Red = IMN; green = PN.

Hyperlipidemia is a common condition in IMN patients, especially in the case of nephrotic syndromes [2]. The lack of knowledge about its role in the pathogenesis and outcome has led it to being out of the spotlight for a long time. The present study demonstrated a significant upregulation in several apolipoproteins, including APOC1, APOB, APOC4 and APOL1, but also in LCAT (Figure 5), suggesting a dysregulation in lipoprotein metabolism that could be partially related to the hyperlipidemia in IMN patients. In line with these attempts, Dong et al. demonstrated that hypercholesterolemia at onset was correlated with glomerular and tubular lesions, glomerular PLA2R deposit, and serum anti-PLA2R titres in a large Chinese cohort. Increased total cholesterol (TC) was shown to be an independent risk factor for IgG deposits, underlying a potential role of hypercholesterolemia in the pathogenesis of MN [22].

Moreover, Wu et al., investigating at the genomic level, showed a strong involvement of lipid metabolism, including cholesterol metabolism and arachidonic acid metabolism, in IMN pathogenesis through key genes, including apolipoprotein C3 (APOC3), cholesteryl ester transfer protein (CEPT), phospholipase A2 group XIIB (PLA2G12B) and also APOA1 and APOB [23]. Moreover, in an animal model of passive Heymann nephritis (that closely mimics IMN at both histological and clinical levels), the presence of intact or fragmented lipoproteins in the glomeruli, with the highest concentration of APOB and APOE within immune deposits, was already demonstrated. The authors stated that the reason for this accumulation can lie in the fact that they may contribute to the formation of intraglomerular lipid peroxidation (LPO) adducts (such as malondialdehyde and 4-hydroxynonenal), thus damaging the glomeruli and promoting the development of proteinuria [24]. Thereby, in this perspective, the presence of the lipoproteins could contribute to the pathogenesis of IMN being responsible for providing fatty acids as substrates for reactive oxygen species (that are produced in part by glomerular epithelial cells via the de novo synthesised NADPH oxido-reductase enzyme complex).

**Figure 6:**
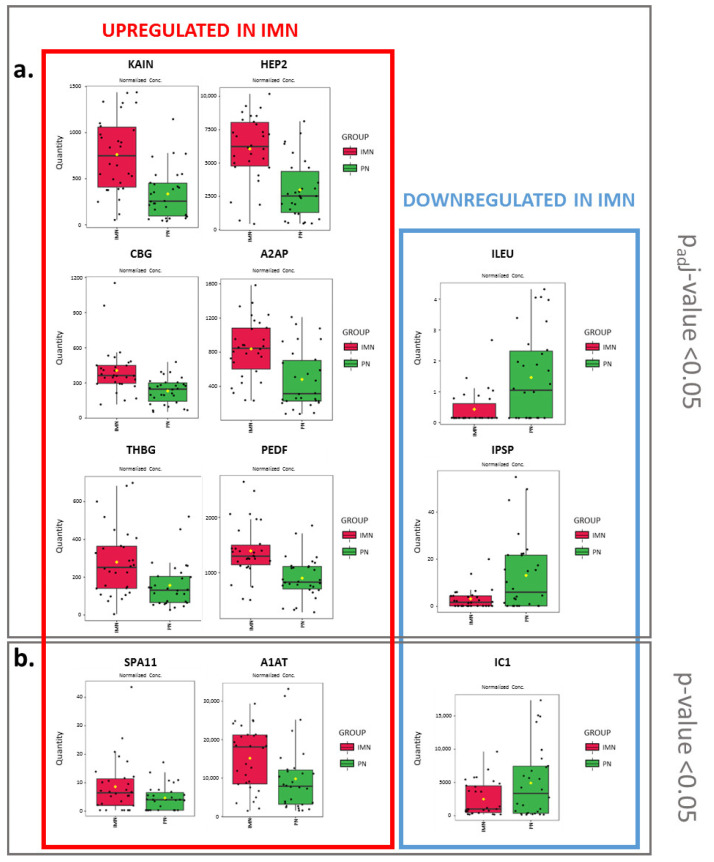
Serpins in IMN. Boxplots of the serpins found to be differentially expressed between IMN and PN groups using high (**a**) (p_adj_-value < 0.05) or lower stringency (**b**) (*p*-value < 0.05). In particular, KAIN, HEP2, THBG, CBG, A2AP, PEDF, SPA11 and A1AT were found to be upregulated in IMN, whereas ILEU, IPSP and IC1 were downregulated. Red = IMN; green = PN.

Among them, KAIN and HEP2 (respectively coded by *SERPINA4* and *SERPIND1* genes) belong to the serpin superfamily. Serpins (serine protease inhibitors) are known to be involved in a number of fundamental biological processes such as blood coagulation, complement activation, fibrinolysis, angiogenesis, inflammation and tumour suppression, and to be expressed in a cell-specific manner [25]. Alongside thyroxine-binding globulin (THBG, coded by *SERPINA7* gene), corticosteroid-binding globulin (CBG, coded by *SERPINA6* gene), alpha-2-antiplasmin (A2AP, coded by *SERPINF2* gene) and pigment epithelium-derived factor (PEDF, coded by *SERPINF1* gene), KAIN and HEP2 were found to be significantly overexpressed in IMN serum samples, while leukocyte elastase inhibitor (ILEU, coded by *SERPINB1* gene) and plasma serine protease inhibitor (IPSP, coded by *SERPINA5* gene) were significantly downregulated (Figure 6a). These observations gain more relevance in this context since the dysregulation of the serpin family has been already associated with nephrotic syndromes and also with IMN. Indeed, Daniel A. Muruve et al. have highlighted an overexpression of SERPINA10, SERPINA4, SERPINC1, SERPINF2 and SERPINF1 in serum samples of MN when compared to serum samples of minimal change disease (MCD), also correlated to the increased risk of thrombotic events in patients with MN [6]. Moreover, Choi et al. used an LC-MS/MS approach to study the urinary proteome of patients affected by MCD, focal segmental glomerulosclerosis (FSGS) and MN and verified the specific overexpression of SERPINA7 in MN samples [9]. Finally, Smith et al. used a MALDI imaging technique in the study of glomerulonephritis biopsies and were able to identify the presence of alpha-1-antitrypsin (A1AT, coded by *SERPINA1* gene) within the cytoplasm of podocyte located in sclerotic glomeruli and suggested a putative role as a marker of podocyte stress (Figure 6b) [11].

An overview of the most critical DEPs found altered in the present study in comparison with literature was summarised in Table 3.

**Figure 7:**
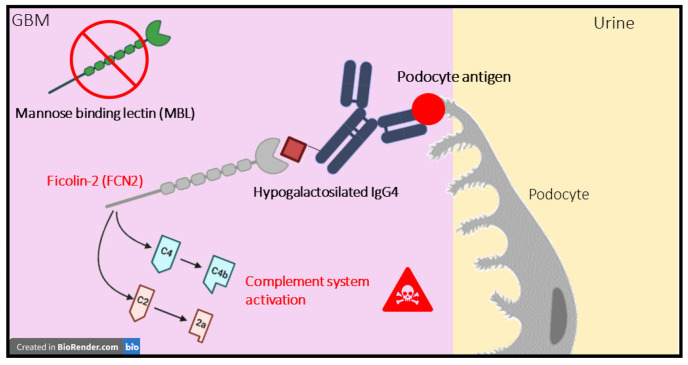
Hypothesis of a possible activated pathway linked to FCN2. Hypothesis of possible activation of the lectin-mediated pathway alternatively sustained by FCN2 in IMN [29,31]. Red = IMN; green = PN. Created with BioRender.com accessed on 20 July 2023.

The functional analysis also pointed out the importance of the *FCN2* gene as a putative key factor in promoting the activation of the complement system in IMN patients. *FCN2* encodes for an N-acetylglucosamine (GlcNAc)-binding lectin referred to as ficolin-2 (or ficolin/P35), which has structural and functional similarities with the mannose binding lectin (MBL) [27]. Caza et al. have previously identified seven new putative biomarkers in MN and membranous lupus nephropathy (MLN) by using an MS approach on biopsy-recovered immunocomplexes, including ficolin-3 (FCN3) [28]. Moreover, Matsushita et al. have demonstrated that the ficolin/P35-MASPs-sMAP complex could be considered a second lectin–serine protease complex for lectin complement pathway activation, thus playing a role in innate immunity [27]. Considering that the typical IgG found in the immunocomplexes does not activate the complement cascade via the classical pathway, but that abundant deposits of complement (including C4) and mannose binding lectin (MBL) are usually observed in the affected tissue, it has been suggested the possibility that the lectin pathway of the complement might be activated via the interaction between MBL and galactose-deficient IgG, thus driving the immune response in the glomerular deposits [10,29,30]. Otherwise, it was observed that IMN can also develop in people with MBL deficiency [31]. Therefore, a possible activation of the lectin-mediated pathway alternatively sustained by FCN2 in IMN could be consistent with our outcome (Figure 7).

Finally, the proteomic tree clustering highlighted two proteins (MRC1 and BTD) which, among all the proteins which resulted deregulated in the two groups, could be possible candidates in helping to discriminate IMN disease from other types of nephropathies (Figure 4).

MRC1 protein, strongly downregulated in IMN vs. PN serum is indeed able by itself to recognise 14 IMN patients over the total of 15 of our dataset, also including 5 PN clusterised with IMN in this step (2 IgAN, 1 FGS, 1 MCD/FSGS and 1 MPGN). *MRC1* gene encodes the transmembrane mannose receptor CD206 which is expressed in macrophages [32]. Macrophages play a critical role in immune surveillance and in kidney homeostasis, and also in the response to acute and chronic kidney injury. Their phenotype can be drastically modulated depending on the nature and duration of renal damage [33]. They are plastic cells able to adapt rapidly to the dynamics of the renal microenvironment and encompass both pro-inflammatory M1 macrophages and the (alternatively activated) M2 subgroup which, on the contrary, has an anti-inflammatory action and can contribute to the resolution of the injury [34]. M2 macrophages, in turn, can be classified into three subpopulations with different phenotypes and roles. In particular, the M2a population (characterised by the transmembrane expression of CD206) is involved in TH2-like immune response, wound healing and tissue fibrosis, and their differentiation is induced by IL-4 and IL-13 [35]. It is noteworthy that CD206+ macrophages are known to be strongly associated with renal fibrosis in human and experimental kidney diseases [36]. Despite several studies reporting the involvement of macrophages in glomerular injury of immune-mediated human glomerulonephritis [37] and the influence of the degree of tubulointerstitial macrophage infiltration in the prognosis of primary MN [38], information about the relationship and the modulation between M2 macrophage subpopulations in IMN glomeruli and the progression and pathogenesis of IMN remains very limited [39]. Very recently, it was observed that peripheral CD206+CD68+ cells in active antineutrophil cytoplasmic antibodies (ANCA) associated glomerulonephritis (AGN) patients were significantly increased in proportion to what measured in remissive patients (*p* < 0.001), healthy controls (*p* < 0.001) and kidney function adjusted controls (*p* < 0.001). Interestingly, the nephropathic control group included mainly primary glomerulonephritis and IgGA nephropathy [40]. *MRC1* is also a paralog of *MRC2*, *LY75* and *PLA2R1*. *PLA2R1* has been well known to encode for the secretory phospholipase A2 receptor which, as mentioned above, is the major antigen involved in the pathogenesis of adult IMN, even if approximately 20–30% of them are negative to anti-PLA2R [41]. It has been shown, by screening the serum from patients with idiopathic membranous nephropathy against human glomerular extracts, that no reactivity is present between IMN serum samples and those three paralogs, an observation that is likely to be congruent to the depletion of this protein in serum of IMN pinpointed in our study [26].

Instead, the BTD protein is an enzyme which is responsible for the cleavage and recycling of biotin [42]. Biotin is a cofactor of the carboxylase enzymes involved in branch-chain amino acid catabolism, gluconeogenesis and the synthesis of fatty acids. People who are affected by biotinidase deficiency have difficulties in recycling biotin from endogenous proteins. Biotinidase deficiency can be developed at different severity grades, depending on the residual enzyme activity. If untreated, neurological and cutaneous symptoms may develop, but also other clinical symptoms can occur (hearing loss, vision problems, developmental delay, hyperventilation, apnoea, stridor and optic atrophy) [43]. Based on our knowledge, there are no studies regarding BTD involvement in IMN disease and this is the first time that this protein seems to be correlated to the specificity of the IMN compared to other nephropathies. Thus, a further evaluation of its possible role could be of interest to better define the molecular setting of this disease.

### Limitations

Some limitations are present in our study, mainly concerning the size of the used cohort. In order to match patients by age and gender, and to select appropriate controls, only 30 individuals were enrolled in the analysis. The choice of a strict sample matching was determined by the need to avoid bias related to confounding factors linked to very diverse distributions of sex and age prevalence and to the not well-balanced control population as normaliser. However, our results find strong confirmation in the literature, both for the proteome and functional aspects as detailed, as undirected positive validation of the observed signatures.

## 4. Materials and Methods

### 4.1. Chemicals and Reagents

The chemicals and reagents used in this work are the following: water (LC-MS grade-LiChrosolv^®^) (Merck KGaA, Darmstadt, Germany), acetonitrile (LC-MS grade-LiChrosolv^®^) (Merck KGaA, Darmstadt, Germany), 2-propanol (LC-MS grade-LiChrosolv^®^) (Merck KGaA, Darmstadt, Germany), ammonium bicarbonate (NH_4_HCO_3_) (Sigma-Aldrich, Buchs, Switzerland), RapiGest^TM^ SF surfactant (Waters, Milford (MA), USA), DL-dithiothreitol (DTT) (Sigma-Aldrich, Buchs, Switzerland), iodoacetamide (IAA) (Sigma-Aldrich, Buchs, Switzerland), trypsin (porcine pancreas) (Sigma-Aldrich, Buchs, Switzerland), trifluoroacetic acid (TFA) (Honeywell SC, Seeelze, Germany), formic acid (FA) LiChropur^®^ (Merck KGaA, Darmstadt, Germany), Pierce^TM^ HeLa protein digest standard (Thermo Scientific^TM^, Waltham, MA, USA) and MMI-L low concentration tuning mix (Agilent Technologies, Santa Clara, CA, USA).

### 4.2. Serum Sample Collection

Serum samples were collected from patients affected by IMN who were positive for PLA2R Ab (15 subjects) in the serum and from patients affected by nephropathy not attributable to IMN but with the same clinical presentation (nephrotic syndrome, PN, 15 subjects), as already described in Chinello C. et al. [10]. All the patients were Caucasian and were enrolled at the time of diagnosis. The control group patients were selected on the bases of similar clinical presentation of membranous nephropathy, which is nephrotic range proteinuria and normal or slightly decreased renal function. Their serum was collected at the institutions where they were diagnosed and they were processed and tested immediately or stored at −80 °C until testing. The diagnosis of IMN and clinical evaluation of the disease activity were performed by the physician in charge of the study in each of the participating nephrology units. The study was approved by the local ethics committee (N. 150/ST/2014, 1 October 2014) and informed consent was obtained from all patients for the treatment of data already collected for routine clinical use. Patient data were anonymously analysed in accordance with the latest version of the Helsinki Declaration of human research ethics.

Blood samples were collected using a 5 mL serum collection tube; after keeping the samples for 30 min at room temperature in an upright position, they were centrifuged for 15 min at 15,000× *g* rpm and then stored at −80 °C until analysis.

Only anti-PLA2R-positive IMN patients were included in the cohort. Anti-PLA2R antibody detection was performed using the commercial human embryonic kidney (HEK)-293 transfected cell-based IIFT (PLA2R IIFT, Euroimmun), as previously described [10].

IMN and PN samples were matched by age (±3 years) and sex, in order to minimise biological variability and to maximise the heterogeneity of the pathological spectrum. Patient data are summarised in Table 1 and Table S3.

### 4.3. Trypsin Digestion

Protein digestion was performed as already reported with few modifications [44]. Briefly, 400 µg of proteins for each sample were diluted with ammonium bicarbonate (NH_4_HCO_3_, Sigma-Aldrich, ≥99.0%, Darmstadt, Germany) buffer solution at 50 mM and with RapiGest^TM^ SF Surfactant (Waters Corporation, Milford, MA, USA) at a final concentration of 0.1%. The proteins were reduced by adding DL-dithiothreitol (DTT) (Sigma-Aldrich, St. Louis, MO, USA, ≥99.5%) at a final concentration of 40 mM and incubated for 45 min at 56 °C; carbamidomethylation reaction was carried out by 55 mM iodoacetamide (IAA) (Sigma-Aldrich, St. Louis, MO, USA) at room temperature for 30 min in the dark.

Trypsin (trypsin from the porcine pancreas, Sigma-Aldrich, St. Louis, MO, USA) was added to the sample for protein digestion at an enzyme/protein ratio of 1:100 and incubated overnight at 37 °C. The digestion was stopped by adding trifluoroacetic acid (TFA) (Honeywell, Seelze, Germany) at a final concentration of 0.5% to reach an acidic pH (<2). RapiGest^TM^ SF surfactant removal was operated by incubating the samples for 30 min at 37 °C followed by centrifugation at 13,000× *g* rpm for 10 min. The supernatant which contained the peptides was finally collected and analysed.

### 4.4. Mass Spectrometry Analysis

For each sample, 400 ng of peptides were injected in duplicate into an Evosep One (Evosep Biosystems, Odense, Denmark) LC system coupled online with a timsTOF fleX^TM^ (Bruker Daltonics, Bremen, Germany) mass spectrometer. The samples were loaded into a disposable trap column, Evotip Pure^TM^ (Evosep Biosystems, Odense, Denmark), following the manufacture protocol. Desalted and concentrated peptides were separated into an analytical 15 cm column (PepSep C18, 15 cm performance column, particle size of 1.5 μm and internal diameter of 150 μm) at a temperature of 40° C. For the separation, a gradient of solvent A (0.1% FA) and solvent B (ACN + 0.1% FA) was used with a gradient of 44 min (30 SPD). The eluted peptides were ionised using a nanoBoosterCaptiveSpray™ (Bruker Daltonics). The mass spectrometer was operated in data-independent acquisition (DIA)-parallel accumulation-serial fragmentation (PASEF) mode. Ions were scanned in positive mode, over an m/z of 100–1700 and a mobility range of 0.85–1.30 V·s/cm^2^. Dry gas flow was 3.0 L/min at 180 °C and capillary voltage was 1400 V. For tandem mass PASEF analysis, the cluster of mono-charged ions was excluded to reduce the complexity of MS2 spectra using the following parameters: *m*/*z* 475–1000 Da and 0.85–1.27 V·s/cm^2^, the estimated cycle time for each PASEF analysis was 0.95 s with a total of 8 cycles using DIA windows of 25 Da.

The mass spectrometer was calibrated for mass and ion mobility accuracy, using a mix of ten standards with a known mass (MMI-L Low Concentration Tuning Mix, Agilent Technologies, Santa Clara, CA, USA). For calibration on the nano-source, three specific lock masses (622.0290 *m*/*z*, 922.0098 *m*/*z* and 1221.9906 *m*/*z*) have been applied on a filter.

### 4.5. Data Processing

Raw data were elaborated by using Spectronaut^TM^ (v.17, https://biognosys.com) following a library-free processing method. A human database (Swissprot, downloaded on 9 June 2022) was used. The parameters were set as follows: trypsin/P as the enzyme, carbamidomethyl (C) as the fixed modifications, acetylation (protein N-term) and oxidation (M) as the variable modifications, 1% FDR at precursor and protein levels. Abundance values were automatically normalised across runs. Proteins were considered identified and quantified only if they had at least one and two significant peptides, respectively.

The statistical analysis was performed on quantified proteins by using the MetaboAnalyst platform (www.metaboanalyst.ca); data were processed with univariate statistics methods (*t*-test), in order to generate volcano plots, boxplots and a hierarchically clustered heatmap.

Volcano plots were performed setting a fold-change (FC) of 1.5 and a *p*-value < 0.05, with or without Benjamini–Hochberg adjustment for multiple comparisons (higher or lower stringency conditions, respectively).

The hierarchical clustered analysis (HCA) was conducted by using default software settings. Data were autoscaled. Euclidean was used as a distance measure, and ward as a clustering method. Heatmap data visualisation showed the top 76 features selected by *t*-test (p_adj_-value < 0.05).

Then, the panel of proteins which resulted differentially expressed (excluding histones to avoid possible bias [44]) was analysed with the open-source R software v.4.1.3 (R Foundation for Statistical Computing, Vienna, Austria); in particular, it was used to perform a classification tree able to select the most impactful proteins that discriminate between IMN and PN patients and to build boxplots of these proteins.

Functional annotations were performed using g:Profiler (https://biit.cs.ut.ee/gprofiler/gost, 30 December 2022) [20].

## 5. Conclusions

Exploring new MN biomarkers has become a research hotspot, especially for the idiopathic forms, whose pathogenic mechanisms are not completely understood [1]. Several authors have tried to define a specific proteomic signature by applying MS approaches to kidney biopsies and urine [9]. However, to the best of our knowledge, no significant untargeted proteomic investigation has been conducted on blood-derived specimens, mainly due to complexity-related issues.

Thus, the present study aimed to investigate possible IMN-specific molecular and functional signatures in undepleted serum samples by the application of a bottom-up nLC-ESI-MS/MS proteomic approach based on the advanced DIA-PASEF acquisition method, which can enable a deeper proteome coverage and comprehensive together with an accurate and reproducible quantitation in order to characterise also the blood hidden proteome.

The application of the DIA-PASEF tailor-made workflow, successfully optimised, unveiled the presence of an IMN-specific panel of 25 over-regulated proteins, including several serpins and lipoprotein-related proteins. A dysregulation of serpins has been already associated with nephrotic syndromes and also with IMN, and could be correlated to the increased risk of thrombotic events usually seen in patients [6]. Additionally, given their implication in multiple biological processes, such as inflammation and complement activation, further investigations are needed to better define their function in IMN pathogenesis. On the other end, our functional enrichment findings reinforce a possible critical role of lipids and lipoproteins in IMN pathogenesis and progression, as already observed in genomic and pre-clinical studies, since they can be considered catalysts for the formation of lipid peroxidation adducts that synergistically contribute to damage the glomeruli [24]. Furthermore, the functional insights of the immune response in IMN patients have enabled us to highlight a possible involvement of ficolin-2 (FCN2) as a novel potential actor in IMN pathogenesis, since (i) it already demonstrated its capability to turn on the lectin-mediated complement pathway, whose activation was shown to be sustained in IMN affected podocytes by the interaction between anti-PLA2R IgG4 and mannose-binding lectin (MBL), and (ii) that IMN can also develop in subjects with MBL deficiency.

In addition, based on a supervised classification tree, two possible candidates (MRC1 and BTD) among the deregulated protein panel showed the ability to distinguish IMN from other nephrotic syndromes with a sensitivity of 93% and a specificity equal to 100%. In particular, the transmembrane mannose receptor CD206 (coded by *MRC1* gene) typically expressed on the surface of macrophages M2a population, and observed as less abundant in IMN vs. PN, is able by itself to detect almost all the IMN patients and its depletion in idiopathic forms seems to find possible molecular contextualization based on the current literature.

Taken together, these results represent a step forward in the comprehension and characterisation of IMN pathogenesis, and once validated in a larger cohort with a proper tailored strategy [45], could pave the way for a proper stratification of IMN patients towards a personalised and targeted therapy.

## Figures and Tables

**Table 1:** Patient detailed list. List of the patients enrolled in the study; for each patient, the diagnosis, group, additional information, gender, age, PLA2R positive or negative and PLA2R titre have been indicated. IMN = idiopathic membranous nephropathy, LMN = lupus membranous nephropathy, FGS = focal glomerulosclerosis, FSGS = focal segmental glomerulosclerosis, MCD = minimal change disease, IgAN = IgA nephropathy, MPGN = membranoproliferative glomerulonephritis, HSP = Henoch–Schönlein purpura.

Sample ID	Diagnosis	Group	Additional Info	Gender	Age	PLA2R pos/neg	PLA2R Titre
IMN v10	IMN	IMN		M	34	pos	1000
IMN v8	IMN	IMN		F	40	pos	320
IMN v14	IMN	IMN		M	40	pos	320
IMN v15	IMN	IMN		F	41	pos	1000
IMN o12	IMN	IMN		F	44	pos	320
IMN v13	IMN	IMN		M	47	pos	1000
IMN v37	IMN	IMN		F	51	pos	1000
IMN v30	IMN	IMN		M	51	pos	320
IMN v22	IMN	IMN		M	54	pos	320
IMN v5	IMN	IMN		M	67	pos	1000
IMN v9	IMN	IMN	moderate nephroangiosclerosis	M	68	pos	320
IMN o1	IMN	IMN		F	70	pos	320
IMN v1	IMN	IMN		F	72	pos	100
IMN v20	IMN	IMN	sclerotic progression	M	73	pos	100
IMN v2	IMN	IMN	declivious oedemas	F	83	pos	320
PN12	Amyloidosis nephropathy	PN		M	35	neg	<10
PN3	MCD/FSGS	PN		F	40	neg	<10
PN8	MCD	PN	IgM deposits	F	40	neg	<10
PN4	MPGN	PN		M	44	neg	<10
PN7	MPGN	PN	IgM deposits	F	45	neg	<10
PN11	MCD	PN		M	47	neg	<10
PN9	Fabry disease	PN		F	49	neg	<10
PN17	FGS	PN		M	51	neg	<10
PN2	FGS	PN	outcome of FGS-Alport like	M	56	neg	<10
PN10	IgAN	PN		M	65	neg	<10
PN19	MCD	PN		F	71	neg	<10
PN14	IgAN	PN		M	71	neg	<10
PN5	FSGS	PN		F	72	neg	<10
PN20	MPGN	PN	Acute renal failure (ARF) with nephrotic syndrome (NS)	M	73	neg	<10
PN1	FGS	PN		F	81	neg	<10

**Table 2:** Differentially expressed protein groups. A list of the 57 differentially expressed protein groups (p_adj_-value < 0.05) identified in the comparison between IMN and PN groups. For each protein group, the protein accession, gene name, protein description and fold change (FC) are reported.

Protein Names	Protein Accessions	Genes	Protein Descriptions	Fold Change (FC)	
**APOC1_HUMAN**	P02654	*APOC1*	Apolipoprotein C-I	12.0	**UPREGULATED IN IMN**
**KV240_HUMAN; KVD40_HUMAN**	A0A087WW87; P01614	*IGKV2-40; IGKV2D-40*	Immunoglobulin kappa variable 2-40; Immunoglobulin kappa variable 2D-40	3.7	
**CRIS2_HUMAN**	P16562	*CRISP2*	Cysteine-rich secretory protein 2	2.8	
**GPX3_HUMAN**	P22352	*GPX3*	Glutathione peroxidase 3	2.6	
**PTGDS_HUMAN**	P41222	*PTGDS*	Prostaglandin-H2 D-isomerase	2.3	
**PCYOX_HUMAN**	Q9UHG3	*PCYOX1*	Prenylcysteine oxidase 1	2.3	
**KAIN_HUMAN**	P29622	*SERPINA4*	Kallistatin	2.3	
**LCAT_HUMAN**	P04180	*LCAT*	Phosphatidylcholine-sterol acyltransferase	2.1	
**HEP2_HUMAN**	P05546	*SERPIND1*	Heparin cofactor 2	2.0	
**APOB_HUMAN**	P04114	*APOB*	Apolipoprotein B-100	2.0	
**PON3_HUMAN**	Q15166	*PON3*	Serum paraoxonase/lactonase 3	2.0	
**SPP24_HUMAN**	Q13103	*SPP2*	Secreted phosphoprotein 24	1.9	
**THBG_HUMAN**	P05543	*SERPINA7*	Thyroxine-binding globulin	1.8	
**CHLE_HUMAN**	P06276	*BCHE*	Cholinesterase	1.8	
**BTD_HUMAN**	P43251	*BTD*	Biotinidase	1.8	
**IL1AP_HUMAN**	Q9NPH3	*IL1RAP*	Interleukin-1 receptor accessory protein	1.8	
**CBG_HUMAN**	P08185	*SERPINA6*	Corticosteroid-binding globulin	1.8	
**A2AP_HUMAN**	P08697	*SERPINF2*	Alpha-2-antiplasmin	1.7	
**FCN2_HUMAN**	Q15485	*FCN2*	Ficolin-2	1.7	
**APOC4_HUMAN**	P55056	*APOC4*	Apolipoprotein C-IV	1.7	
**PRG4_HUMAN**	Q92954	*PRG4*	Proteoglycan 4	1.6	
**APOL1_HUMAN**	O14791	*APOL1*	Apolipoprotein L1	1.6	
**PCOC1_HUMAN**	Q15113	*PCOLCE*	Procollagen C-endopeptidase enhancer 1	1.6	
**PEDF_HUMAN**	P36955	*SERPINF1*	Pigment epithelium-derived factor	1.5	
**FETUB_HUMAN**	Q9UGM5	*FETUB*	Fetuin-B	1.5	
**IGL1_HUMAN**	P0DOX8		Immunoglobulin lambda-1 light chain	0.7	**DOWNREGULATED IN IMN**
**LV147_HUMAN**	P01700	*IGLV1-47*	Immunoglobulin lambda variable 1-47	0.7	
**LV39_HUMAN**	A0A075B6K5	*IGLV3-9*	Immunoglobulin lambda variable 3-9	0.7	
**KPYM_HUMAN**	P14618	*PKM*	Pyruvate kinase PKM	0.6	
**PERM_HUMAN**	P05164	*MPO*	Myeloperoxidase	0.6	
**IGG1_HUMAN**	P0DOX5		Immunoglobulin gamma-1 heavy chain	0.6	
**IGA2_HUMAN**	P0DOX2		Immunoglobulin alpha-2 heavy chain	0.6	
**LYSC_HUMAN**	P61626	*LYZ*	Lysozyme C	0.6	
**ENOA_HUMAN**	P06733	*ENO1*	Alpha-enolase	0.5	
**IGHG3_HUMAN**	P01860	*IGHG3*	Immunoglobulin heavy constant gamma 3	0.5	
**KV37_HUMAN**	A0A075B6H7	*IGKV3-7*	Probable non-functional immunoglobulin kappa variable 3-7	0.5	
**LV140_HUMAN**	P01703	*IGLV1-40*	Immunoglobulin lambda variable 1-40	0.5	
**LV861_HUMAN**	A0A075B6I0	*IGLV8-61*	Immunoglobulin lambda variable 8-61	0.5	
**GRN_HUMAN**	P28799	*GRN*	Progranulin	0.5	
**IGLC3_HUMAN**	P0DOY3	*IGLC3*	Immunoglobulin lambda constant 3	0.5	
**LV316_HUMAN**	A0A075B6K0	*IGLV3-16*	Immunoglobulin lambda variable 3-16	0.5	
**LV310_HUMAN**	A0A075B6K4	*IGLV3-10*	Immunoglobulin lambda variable 3-10	0.5	
**PROF1_HUMAN**	P07737	*PFN1*	Profilin-1	0.5	
**ELNE_HUMAN**	P08246	*ELANE*	Neutrophil elastase	0.5	
**G3P_HUMAN**	P04406	*GAPDH*	Glyceraldehyde-3-phosphate dehydrogenase	0.5	
**ACTB_HUMAN**	P60709	*ACTB*	Actin, cytoplasmic 1	0.5	
**ZYX_HUMAN**	Q15942	*ZYX*	Zyxin	0.5	
**MRC1_HUMAN**	P22897	*MRC1*	Macrophage mannose receptor 1	0.4	
**DEF3_HUMAN**	P59666	*DEFA3*	Neutrophil defensin 3	0.4	
**COR1A_HUMAN**	P31146	*CORO1A*	Coronin-1A	0.4	
**ILEU_HUMAN**	P30740	*SERPINB1*	Leukocyte elastase inhibitor	0.3	
**KV139_HUMAN; KVD39_HUMAN**	P01597; P04432	*IGKV1-39; IGKV1D-39*	Immunoglobulin kappa variable 1-39; Immunoglobulin kappa variable 1D-39	0.3	
**H2A1_HUMAN; H2A1D_HUMAN; H2A2C_HUMAN; H2A2A_HUMAN; H2A1H_HUMAN; H2A1J_HUMAN; H2AJ_HUMAN**	P0C0S8; P20671; Q16777; Q6FI13; Q96KK5; Q99878; Q9BTM1	*H2AC11; H2AC7; H2AC20; H2AC18; H2AC12; H2AC14; H2AJ*	Histone H2A type 1; Histone H2A type 1-D; Histone H2A type 2-C; Histone H2A type 2-A; Histone H2A type 1-H; Histone H2A type 1-J; Histone H2A.J	0.3	
**H2B1K_HUMAN; H2BFS_HUMAN; H2B1D_HUMAN; H2B1C_HUMAN; H2B2F_HUMAN; H2B1H_HUMAN; H2B1N_HUMAN; H2B1M_HUMAN; H2B1L_HUMAN**	O60814; P57053; P58876; P62807; Q5QNW6; Q93079; Q99877; Q99879; Q99880	*H2BC12; H2BC12L; H2BC5; H2BC4; H2BC18; H2BC9; H2BC15; H2BC14; H2BC13*	Histone H2B type 1-K; Histone H2B type F-S; Histone H2B type 1-D; Histone H2B type 1-C/E/F/G/I; Histone H2B type 2-F; Histone H2B type 1-H; Histone H2B type 1-N; Histone H2B type 1-M; Histone H2B type 1-L	0.3	
**IPSP_HUMAN**	P05154	*SERPINA5*	Plasma serine protease inhibitor	0.2	
**H14_HUMAN; H13_HUMAN; H12_HUMAN**	P10412; P16402; P16403	*H1-4; H1-3; H1-2*	Histone H1.4; Histone H1.3; Histone H1.2	0.2	
**TYB4_HUMAN**	P62328	*TMSB4X*	Thymosin beta-4	0.1	

**Table 3:** Overview of DEPs already reported altered in renal diseases.

Present Study		Literature Evidence					
Protein	UP/DOWN	Disease	Sample	UP/DOWN	Technique	Reference	
APOB	UP	MN	Renal tissue	UP	Illumina^®^ Whole-Genome Gene Expression Direct Hybridization Assay system	Wu et al. [23]	Lipoprotein-related proteins
		Passive Heymann nephritis model	Renal tissue	UP	Immunohistochemistry	Exner et al. [24]	
KAIN	UP	MN vs. MCD	Serum	UP	Quantitave SOMAscan proteomics	Muruve et al. [6]	Serpins
A2AP	UP	MN vs. MCD	Serum	UP	Quantitave SOMAscan proteomics	Muruve et al. [6]	
THGB	UP	MN	Urine	UP	LC-MS/MS	Choi et al. [9]	
PEDF	UP	MN vs. MCD	Serum	UP	Quantitave SOMAscan proteomics	Muruve et al. [6]	
A1AT	UP	IMN	Renal tissue	UP	MALDI-imaging	Smith et al. [11]	
MRC1	DOWN	IMN	Serum	DOWN	Immunohistochemistry	Tomas et al. [26]	

## Data Availability

The data presented in this study are available in a proteomic public repository (https://massive.ucsd.edu/ProteoSAFe/static/massive.jsp accessed on 29 June 2023) accessible at the following links ftp://massive.ucsd.edu/MSV000092309/; https://proteomecentral.proteomexchange.org/cgi/GetDataset?ID=PXD043405 [46].

## Supplementary Materials

The supporting information can be downloaded at: https://www.mdpi.com/article/10.3390/ijms241411756/s1.
