# Supplementary material for: Towards the Definition of the Molecular Hallmarks of Idiopathic Membranous Nephropathy in Serum Proteome: A DIA-PASEF Approach
Source: Int J Mol Sci. 2023 Jul 21;24(14):11756. doi: 10.3390/ijms241411756 (PMC10380405; doi:10.3390/ijms241411756)
Supplement: Supplementary file 1 [file ijms-24-11756-s001.zip › Supplementary Figures and Tables.pdf]

SUPPLEMENTARY FIGURES AND TABLES

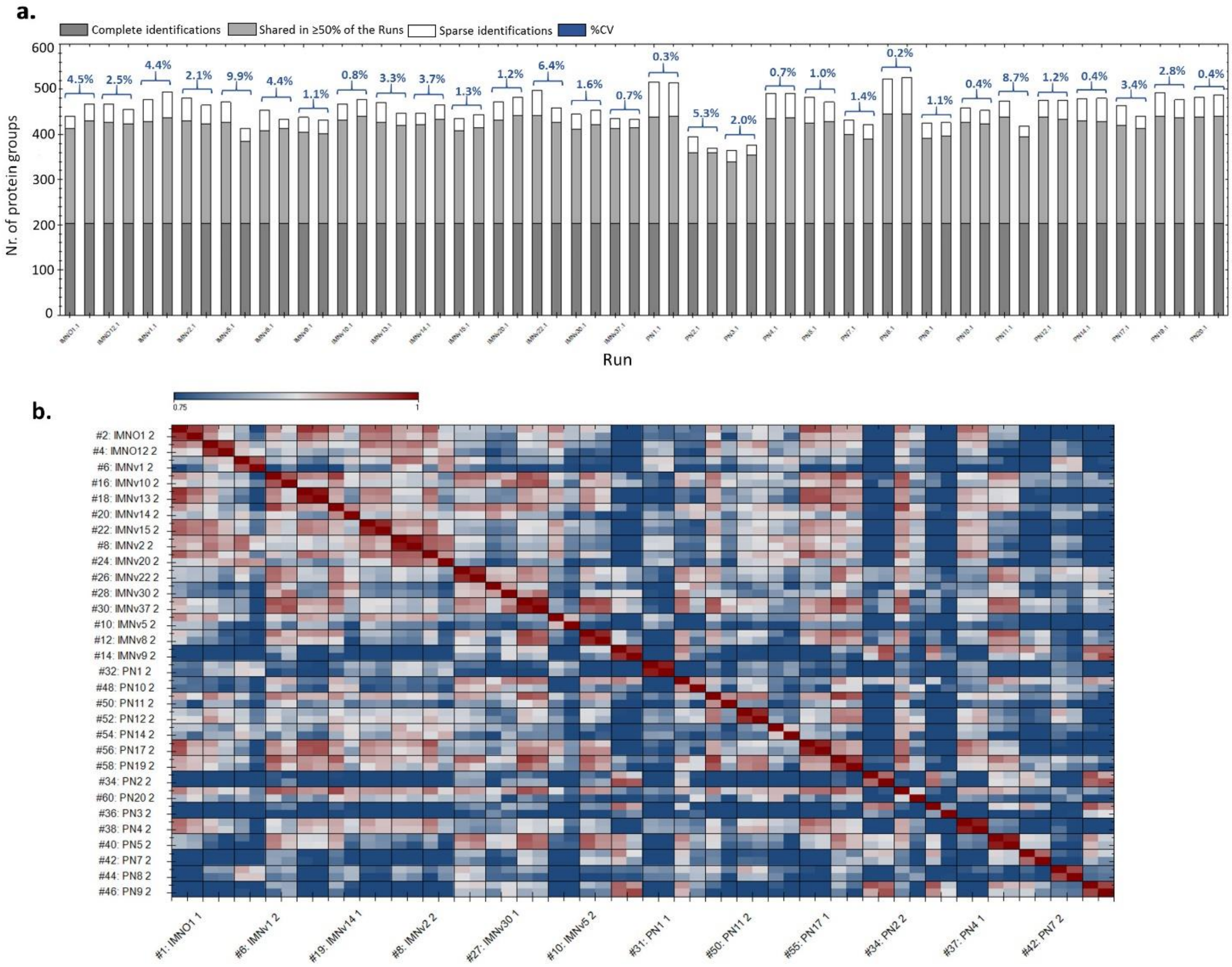

Figure S1. a. **Total number of protein groups identified for each run.** In detail, 203 protein groups are identified in all runs (Complete identification, dark grey). Protein groups shared in ≥50% of the runs or in ≤50% of the runs (Sparse identifications) are represented in light grey and white respectively. **b. Sample correlation matrix.** Red corresponds to maximum correlation (1), while blue corresponds to 0.75 correlation.

Table S1. List of the protein groups identified. List of the protein groups identified, for each protein group is indicated the gene name, the protein description, the protein name, the molecular weight, the number of stripped sequences identified, the p-value and the Q-value. The proteins detected in at least 70% of the runs were highlighted in green.

Table S2. List of the protein groups and peptides identified. List of the peptides identified for each protein group. In blue are highlighted peptides belonging to down-regulated proteins in IMN, while in red are highlighted peptides belonging to up-regulated proteins in IMN. For each protein group is also indicated the gene name, the protein description, the protein name, the molecular weight, the number of stripped sequences identified and the relative normalised abundancies for each run.

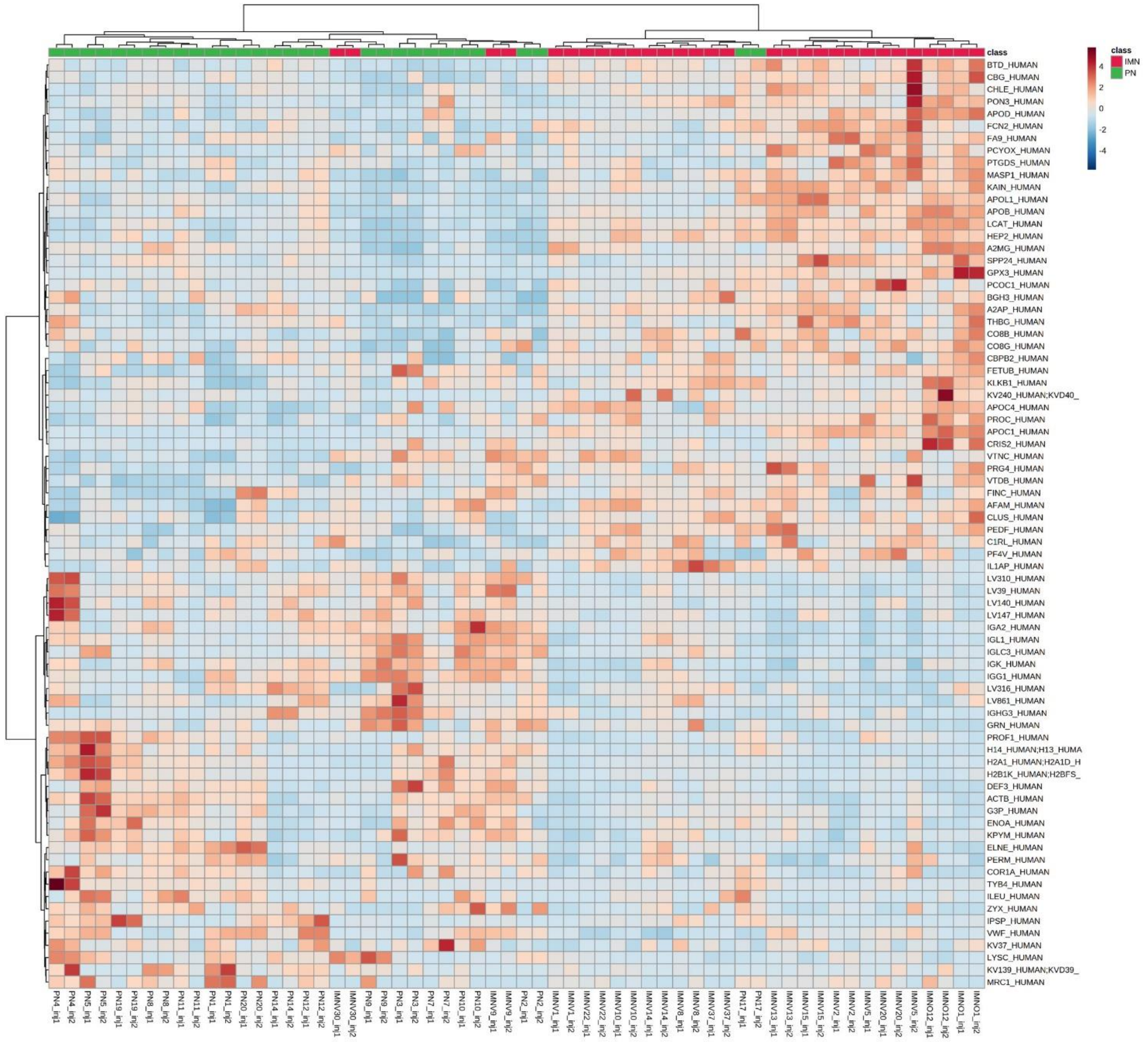

Figure S2. Hierarchical cluster analysis (HCA) with heatmap visualization. The heatmap shows the top 76 features selected by T-test. The data were autoscaled; Euclidean was used as a distance measure, and Ward as a clustering method.

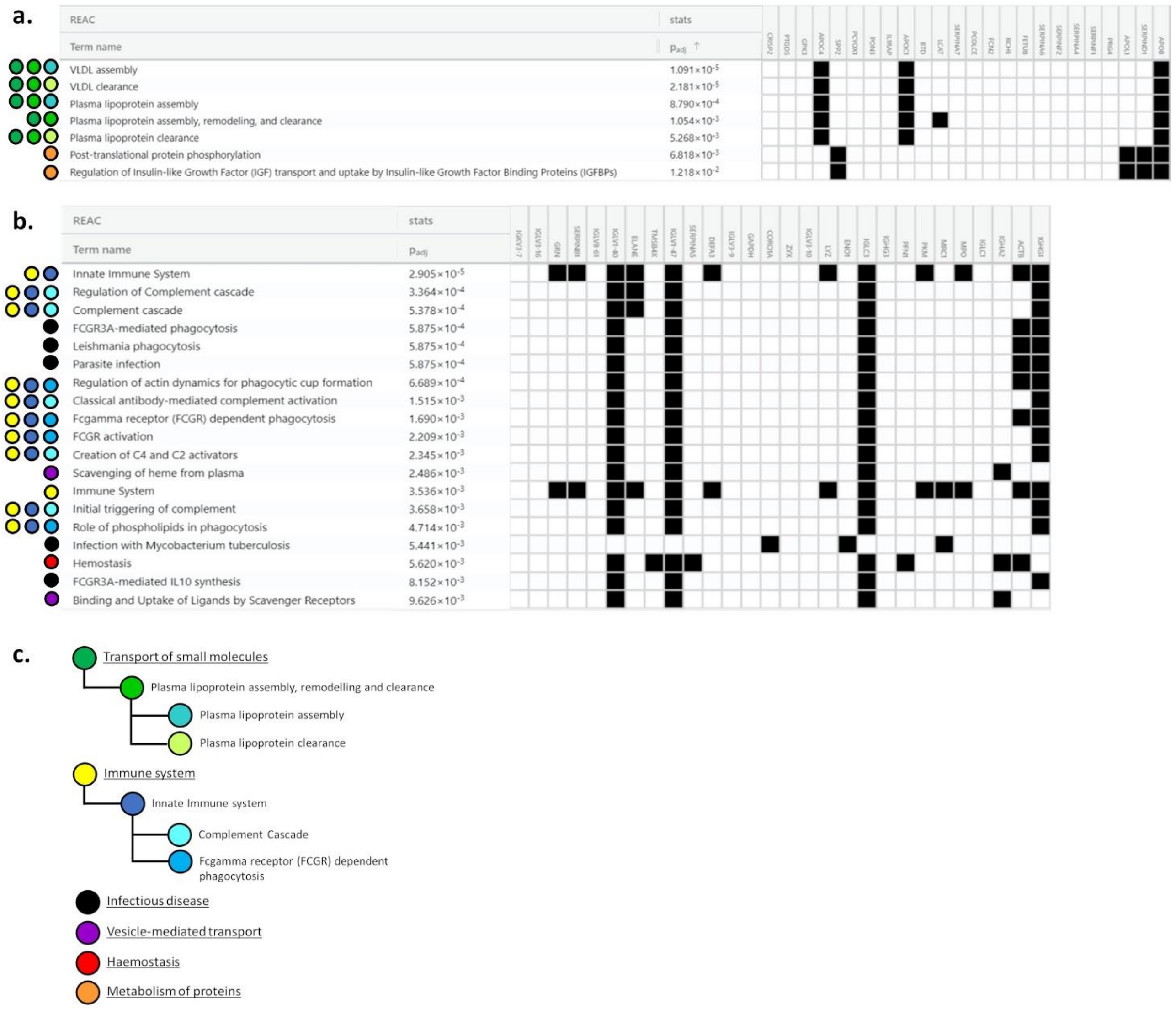

Figure S3. a. Biological processes enriched in the upregulated subset of protein groups. The table shows the REACTOME pathways enriched for the 25 protein groups upregulated in IMN. On the left, each functional signature has been categorised based on the hierarchical pathways clusterization reported in REACTOME database (see c.) using g:Profiler [20]. **b. Biological processes enriched in the downregulated subset of protein groups**. The table shows the REACTOME pathways enriched for the 32 protein groups downregulated in IMN. On the left, each functional signature has been categorised based on the hierarchical pathways clusterization reported in REACTOME database (see c.) using g:Profiler [20]. **c. Legend of the hierarchical pathway clusterization reported in REACTOME database.**

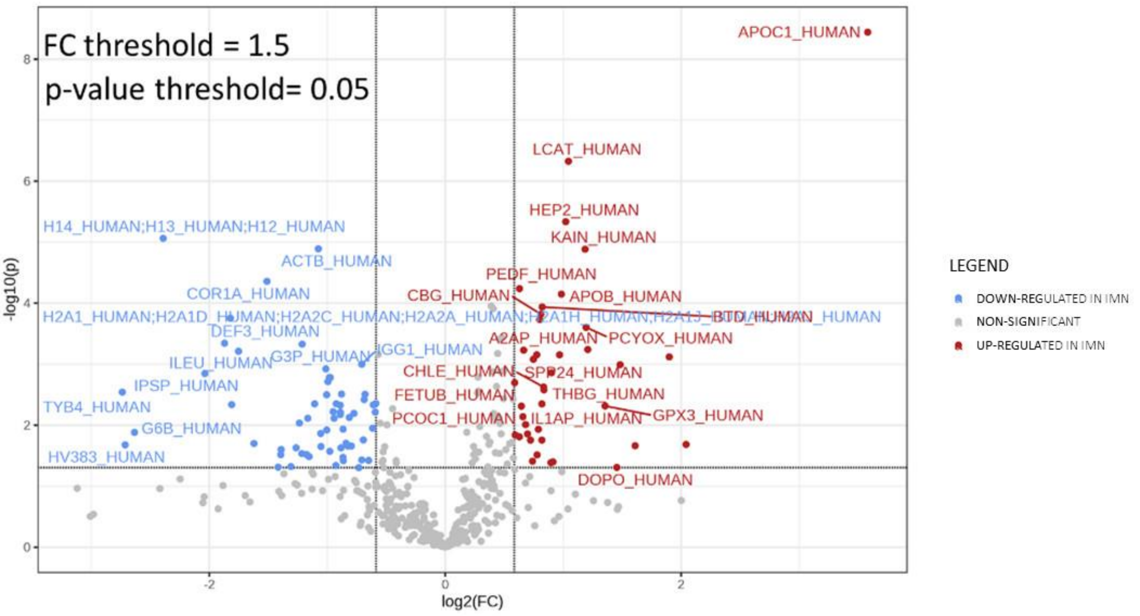

Figure S4. Volcano plot (p-value not adjusted). Differentially expressed proteins between IMN and PN groups. FC is set to 1.5 and non-adjusted p-value at least <0.05. Blue = downregulated proteins in IMN; Red = upregulated proteins in IMN; Grey = nonsignificant proteins**.**

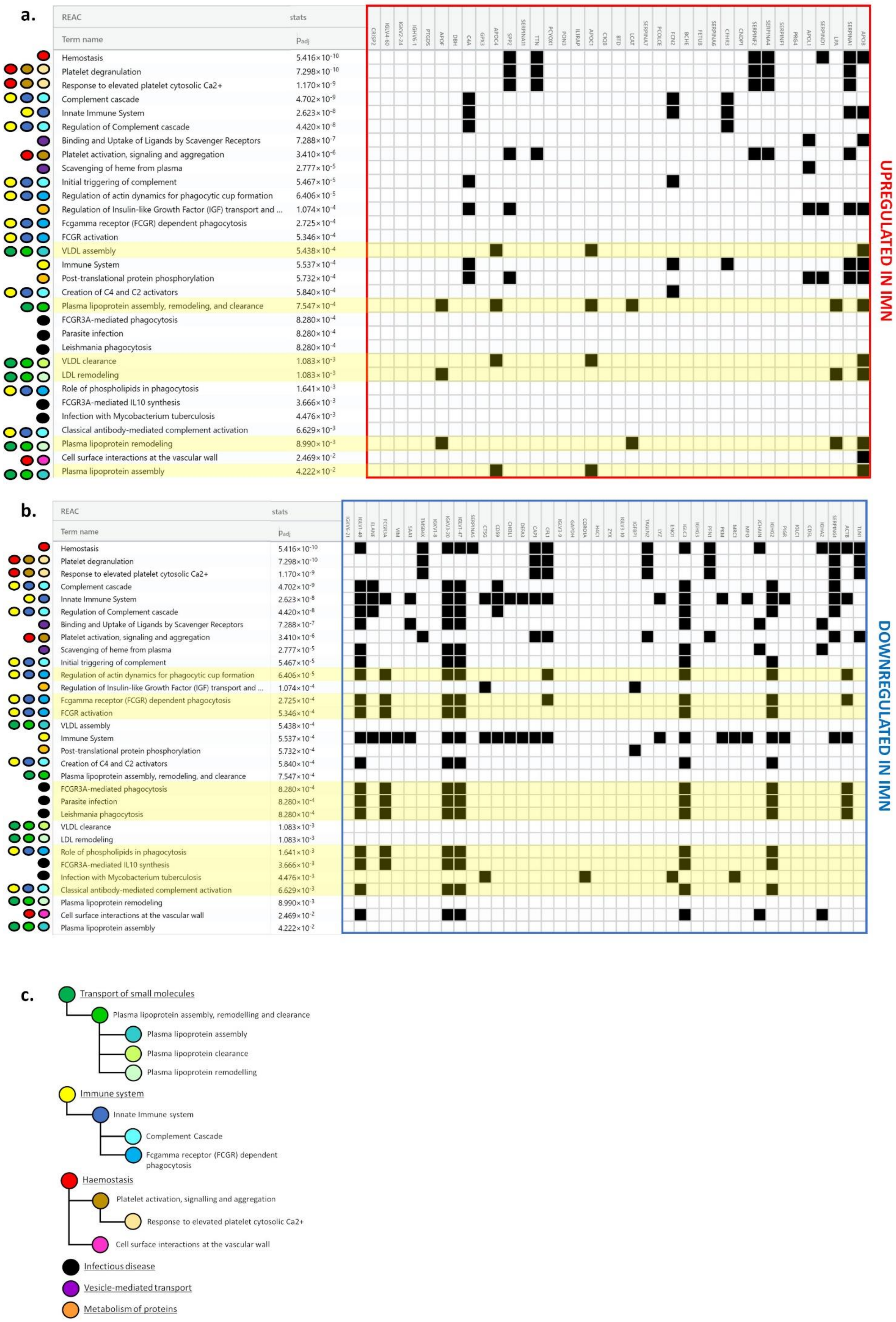

a.
REAC
stats
Term name
Padj
Hemostasis 5.416×10-10
Platelet degranulation 7.298×10-10
Response to elevated platelet cytosolic Ca2+ 1.170×10-9
Complement cascade 4.702×10-9
Innate Immune System 2.623×10-8
Regulation of Complement cascade 4.420×10-8
Binding and Uptake of Ligands by Scavenger Receptors 7.288×10-7
Platelet activation, signaling and aggregation 3.410×10-6
Scavenging of heme from plasma 2.777×10-5
Initial triggering of complement 5.467×10-5
Regulation of actin dynamics for phagocytic cup formation 6.406×10-5
Regulation of Insulin-like Growth Factor (IGF) transport and ... 1.074×10-4
Fcgamma receptor (FCGR) dependent phagocytosis 2.725×10-4
FCGR activation 5.346×10-4
VLDL assembly 5.438×10-4
Immune System 5.537×10-4
Post-translational protein phosphorylation 5.732×10-4
Creation of C4 and C2 activators 5.840×10-4
Plasma lipoprotein assembly, remodeling, and clearance 7.547×10-4
FCGR3A-mediated phagocytosis 8.280×10-4
Parasite infection 8.280×10-4
Leishmania phagocytosis 8.280×10-4
VLDL clearance 1.083×10-3
LDL remodeling 1.083×10-3
Role of phospholipids in phagocytosis 1.641×10-3
FCGR3A-mediated IL10 synthesis 3.666×10-3
Infection with Mycobacterium tuberculosis 4.476×10-3
Classical antibody-mediated complement activation 6.629×10-3
Plasma lipoprotein remodeling 8.990×10-3
Cell surface interactions at the vascular wall 2.469×10-2
Plasma lipoprotein assembly 4.222×10-2
UPREGULATED IN IMN
b.
DOWNREGULATED IN IMN
c.
Transport of small molecules
Plasma lipoprotein assembly, remodelling and clearance
Plasma lipoprotein assembly
Plasma lipoprotein clearance
Plasma lipoprotein remodelling
Immune system
Innate Immune system
Complement Cascade
Fcgamma receptor (FCGR) dependent phagocytosis
Haemostasis
Platelet activation, signalling and aggregation
Response to elevated platelet cytosolic Ca2+
Cell surface interactions at the vascular wall
Infectious disease
Vesicle-mediated transport
Metabolism of proteins

Figure S5. a. Biological processes enriched in the upregulated subset of protein groups identified with lower stringency. The table shows the REACTOME pathways enriched for the 39 protein groups upregulated in IMN. On the left, each functional signature has been categorised based on the hierarchical pathways clusterization reported in REACTOME database (see c.) using g:Profiler [20]. Pathways identified independently from the statistical stringency are highlighted in yellow. **b. Biological processes enriched in the downregulated subset of protein groups identified with lower stringency**. The table shows the REACTOME pathways enriched for the 61 protein groups upregulated in PN (downregulated in IMN). On the left, each functional signature has been categorised based on the hierarchical pathways clusterization reported in REACTOME database (see c.) using g:Profiler [20]. Pathways identified independently from the statistical stringency are highlighted in yellow. **c. Legend of the hierarchical pathway clusterization reported in REACTOME database.**

Table S3. Patient supplementary information. Supplementary information about IMN patients: for each patient urine protein, serum protein, serum albumin and serum creatinine concentrations and therapy have been indicated.
