# Supplementary figures and images for: Towards the Definition of the Molecular Hallmarks of Idiopathic Membranous Nephropathy in Serum Proteome: A DIA-PASEF Approach
Source: Int J Mol Sci. 2023 Jul 21;24(14):11756. doi: 10.3390/ijms241411756 (PMC10380405; doi:10.3390/ijms241411756)

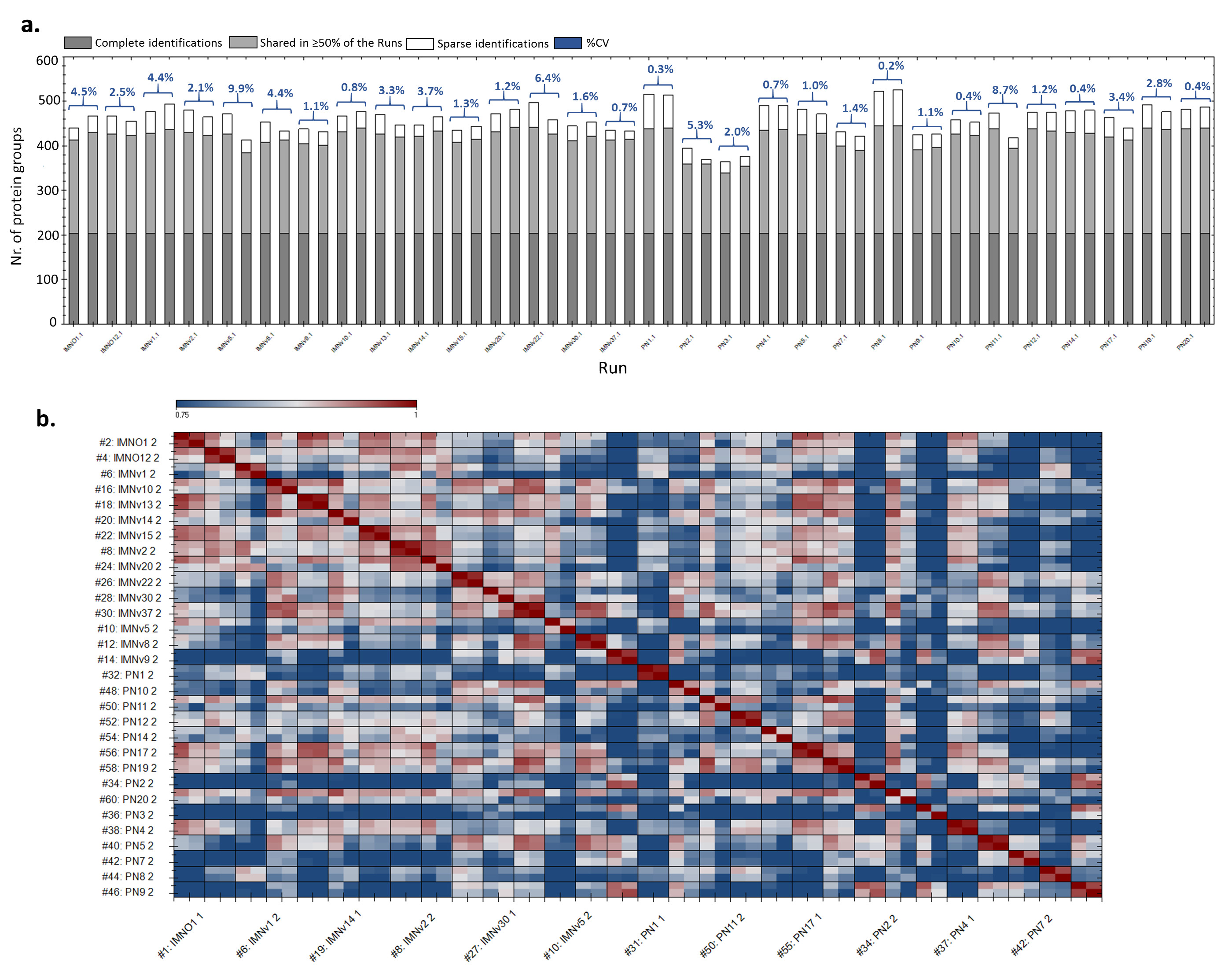

Supplement: Supplementary file 1 [file ijms-24-11756-s001.zip › Figure S1.jpeg]

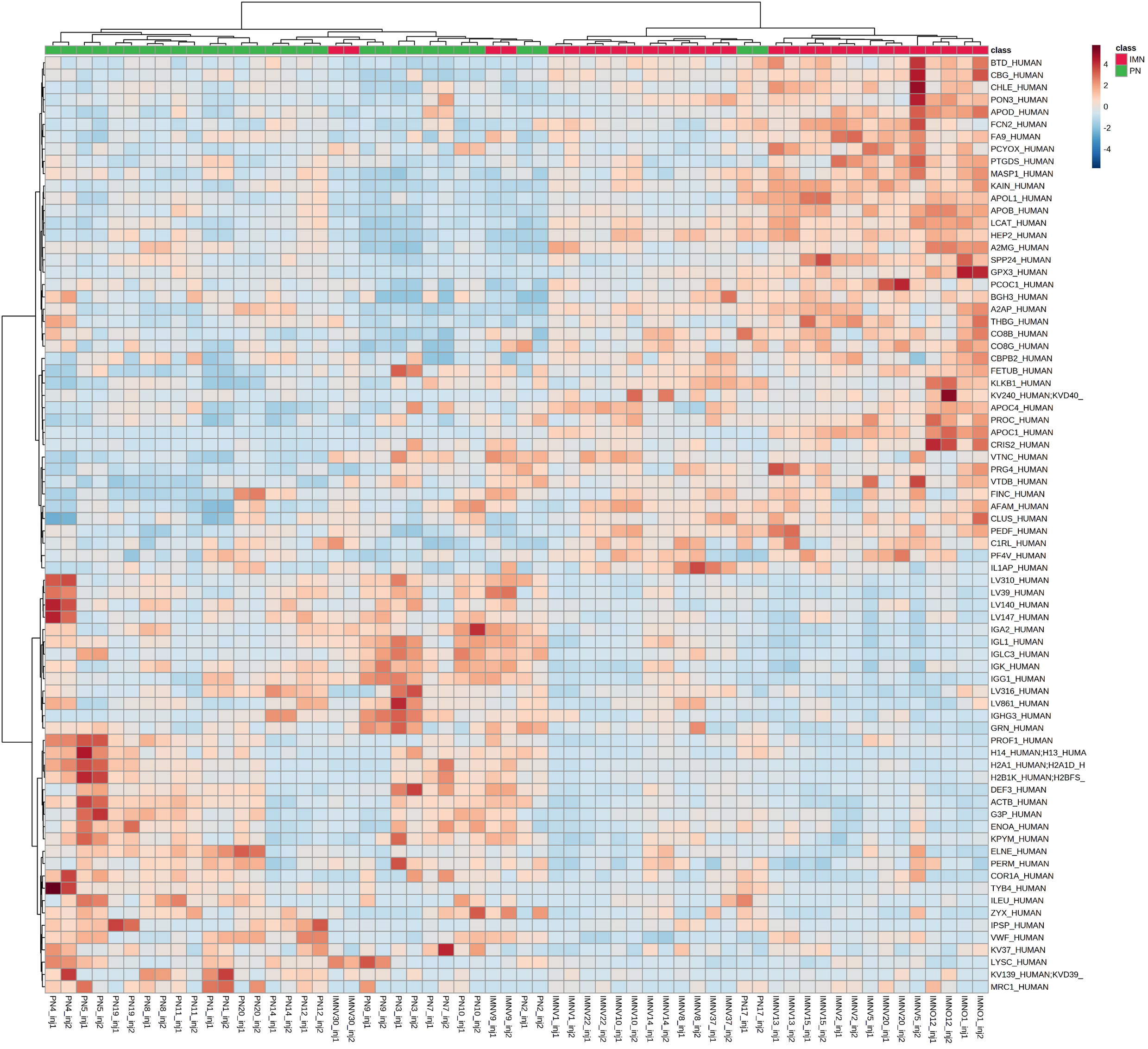

Supplement: Supplementary file 1 [file ijms-24-11756-s001.zip › Figure S2.jpeg]

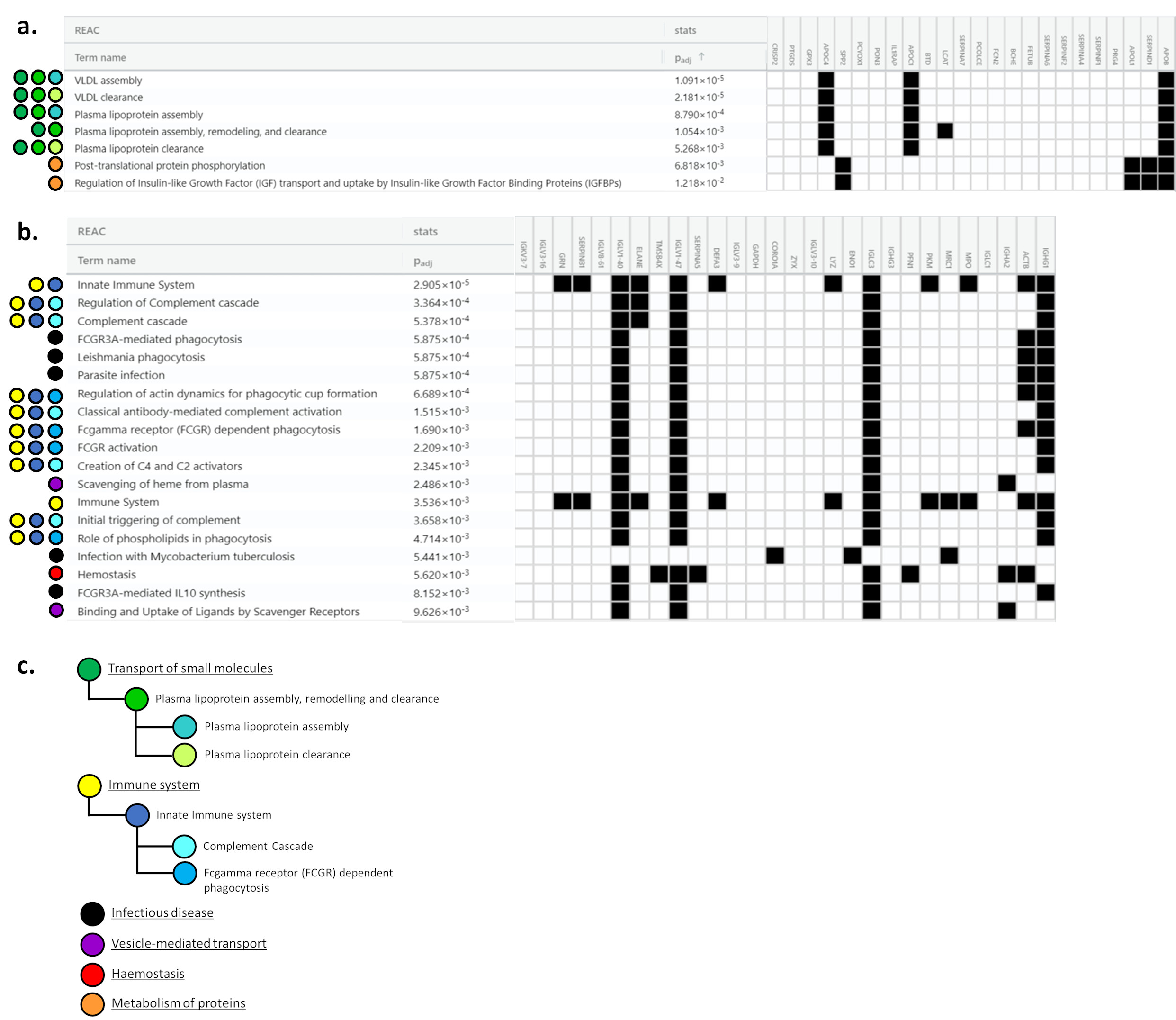

Supplement: Supplementary file 1 [file ijms-24-11756-s001.zip › Figure S3.jpeg]

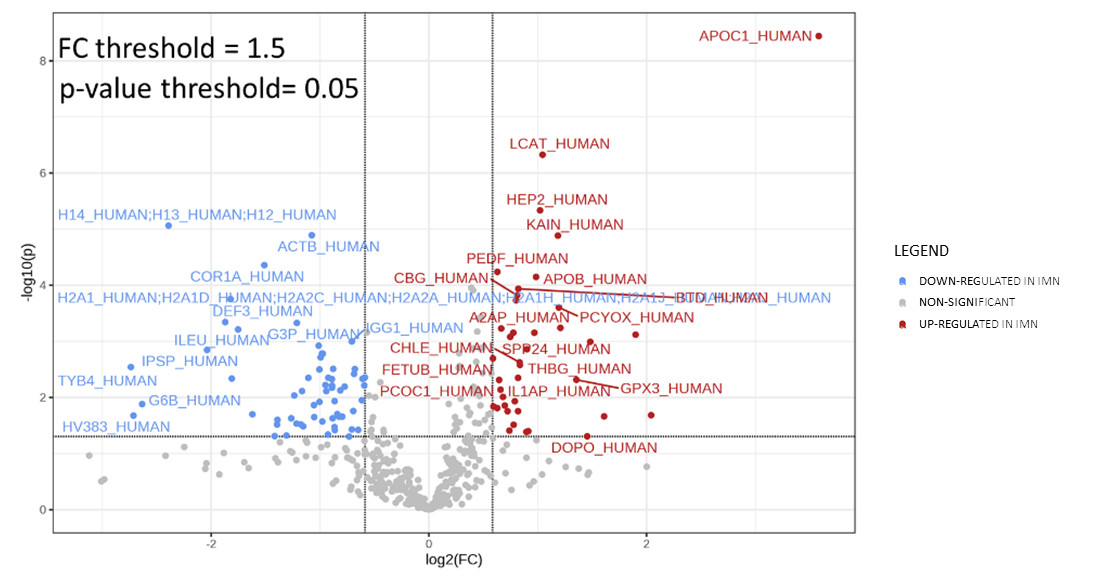

Supplement: Supplementary file 1 [file ijms-24-11756-s001.zip › Figure S4.jpeg]

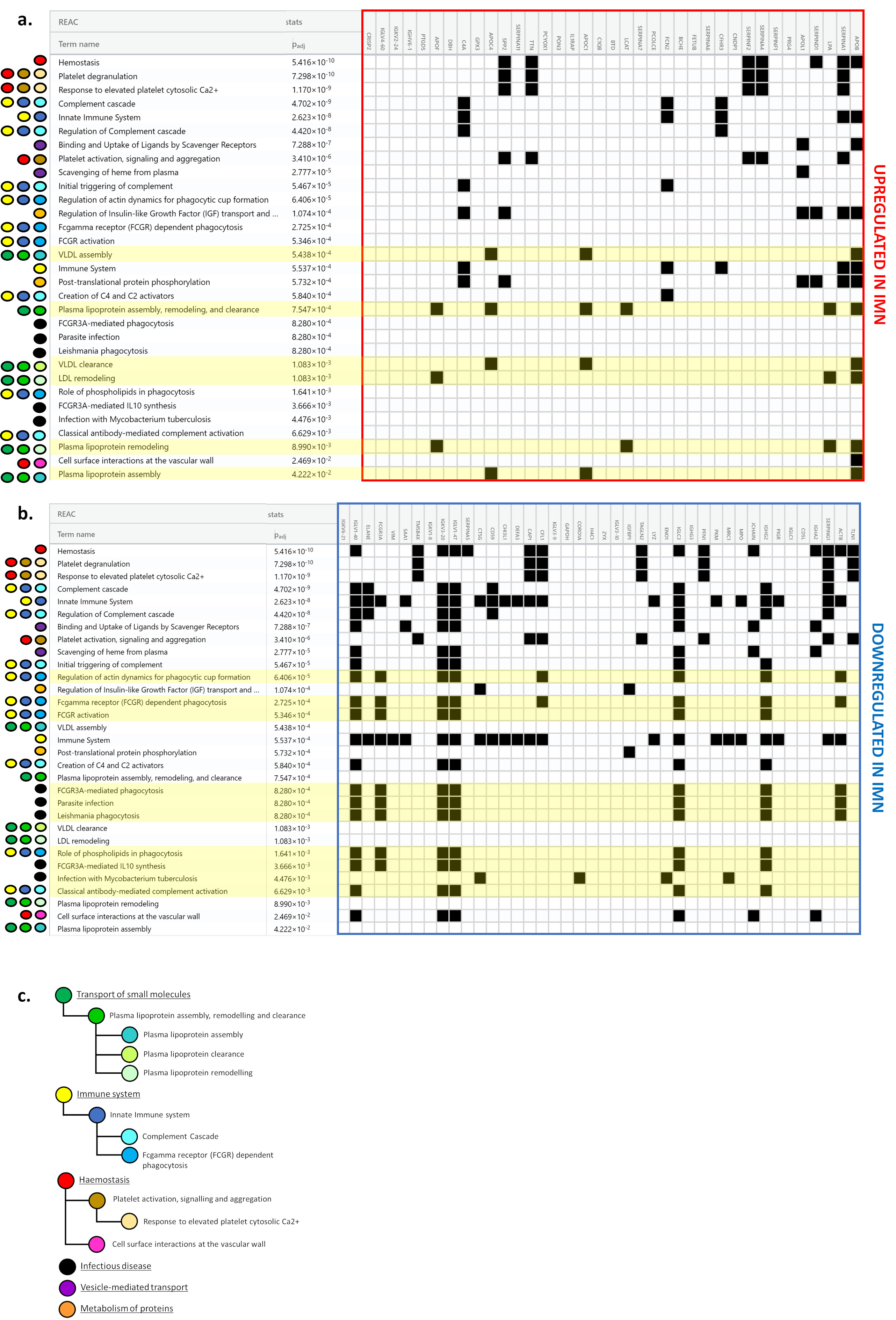

Supplement: Supplementary file 1 [file ijms-24-11756-s001.zip › Figure S5.jpeg]
